# Research Progress on *TRPA1* in Diseases

**DOI:** 10.1007/s00232-023-00277-x

**Published:** 2023-04-11

**Authors:** Jiajing Li, Hongfei Zhang, Qian Du, Junyu Gu, Jiangbo Wu, Qi Liu, Zhuo Li, Ting Zhang, Jingyu Xu, Rui Xie

**Affiliations:** Department of Gastroenterology, Digestive Disease Hospital, Affiliated Hospital of Zunyi Medical University, Zunyi, 563000 China

**Keywords:** *TRPA1*, Ion channel, Ca^2+^, Various systems in the body

## Abstract

For a long time, the physiological activity of *TRP* ion channels and the response to various stimuli have been the focus of attention, and the physiological functions mediated by ion channels have subtle links with the occurrence of various diseases. Our group has been engaged in the study of ion channels. In recent years, the report rate of *TRPA1*, the only member of the *TRPA* subfamily in the newly described *TRP* channel, has been very high. *TRPA1* channels are not only abundantly expressed in peptidergic nociceptors but are also found in many nonneuronal cell types and tissues, and through the regulation of Ca^2+^ influx, various neuropeptides and signaling pathways are involved in the regulation of nerves, respiration, circulation, and various diseases and inflammation throughout the body. In this review, we mainly summarize the effects of *TRPA1* on various systems in the body, which not only allows us to have a more systematic and comprehensive understanding of *TRPA1* but also facilitates more in-depth research on it in the future.

**Graphical Abstract:**

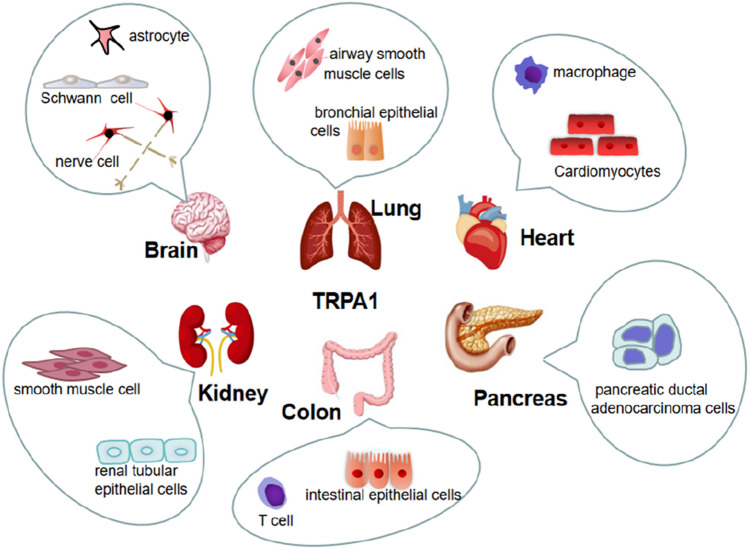

## *TRPA1* introduction

The *TRP* gene was first identified in *Drosophila melanogaster* in the late 1960s (Li 2017). The first human homolog was reported in 1995 (Li 2017). There are seven families of *TRPs: TRPC, TRPV, TRPM, TRPA, TRPP, TRPML*, and *TRPN* (Li 2017). *TRP* channels often affect cellular function by regulating protein expression levels, membrane excitability, and intracellular calcium levels, as cardiac and neuronal disorders are associated with aberrant *TRP* function (Souza Monteiro de Araúja et al. 2020a).

**Fig. 1:**
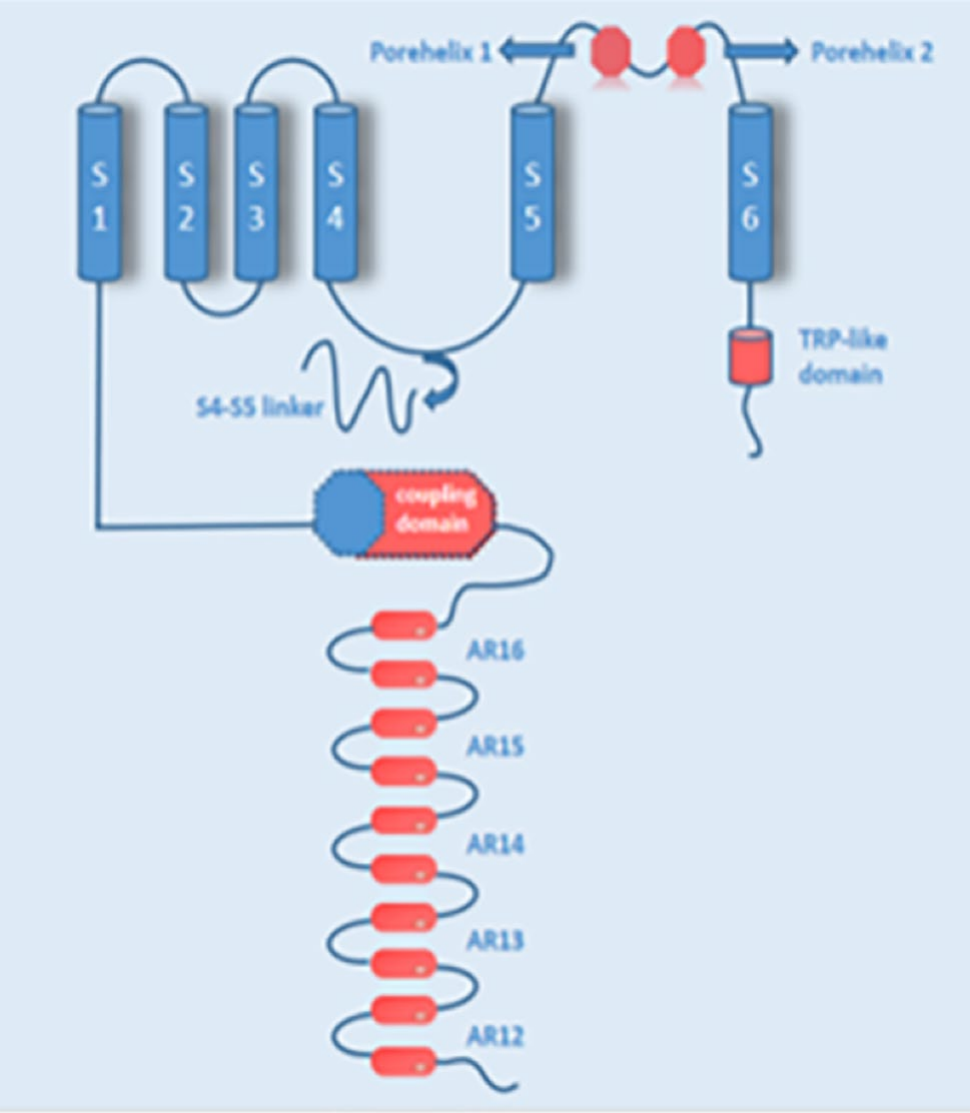
Structure of the TRPA1 channel. TRPA1 has a tetrameric structure, and each subunit contains six transmembrane domains (S1-S6), intracellular NH2- and COOH-termini, and several ankyrin repeats

**Fig. 2:**
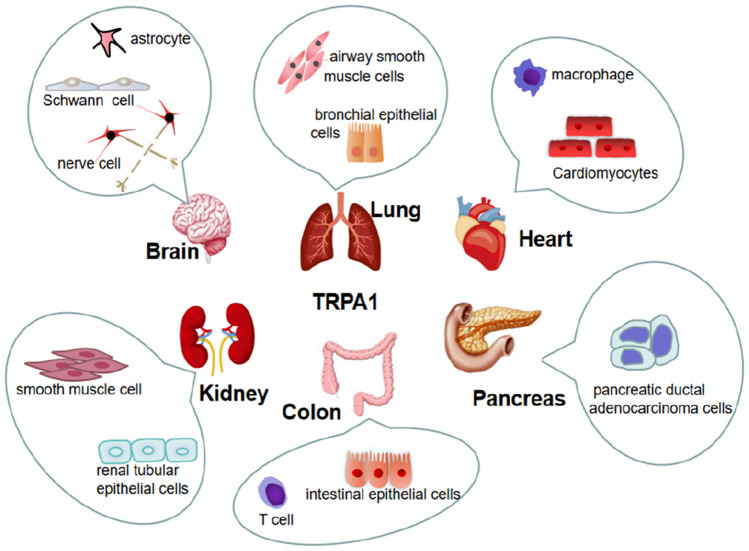
Expression of TRPA1 in the nervous, respiratory, cardiovascular, digestive, and urinary systems. Nervous system mainly expressed in small diameter C or Aδ fibers of sensory ganglia, including DRG, 99 trigeminal, and tubercle ganglia. Respiratory System expressed in trachea, bronchi, and alveolar epithelial cells. Circulatory system expressed in vascular endothelium, cardiomyocytes, and cardiac fibroblasts. Digestive system expressed in enterochromaffin cells in the gut, pancreatic beta cells. Urinary system expressed in renal tubular epithelial cells, urothelial, and smooth muscle cells in the bladder mucosa. Skin expressed in melanocytes

The ankyrin repeat channel *TRPA1*, a member of the *TRP* family, was first cloned in 1999, and in recent years has received much attention owing to its functional diversity as a signal sensor of stimuli and cell damage and its important role in many different diseases (Nilius et al. 2012). The development of various functions is inseparable from its special structure (Fig. 1), such as the elongated ankyrin repeat domain (ARD 14–18), which controls protein‒protein interactions, as well as channel insertion and regulation in the plasma membrane. In addition, it is much more permeable to Ca^2+^ than other *TRPs* (Souza Monteiro de Araújo et al. 2020b). From the cytoplasmic point of view, *TRPA1* is tightly regulated by Ca^2+^ (Sura et al. 2012). It has been proposed that an EF-hand located within the N-terminus of *TRPA1* mediates Ca^2+^ activation (Doerner et al. 2007; Zurborg et al. 2007), another putative Ca^2+^-binding domain is formed by residues E1077, D1080, D1081, and D1082 in the distal COOH-terminal region, the conserved acidic motif at the C-terminal is actively involved in the regulation of *TRPA1* by Ca^2+^ (Sura et al. 2012). A highly conserved structural motif in *TRPA1* is a key site for intracellular Ca^2+^ elevation caused by Ca^2+^ storage (Hu et al. 2021). *TRPA1* can induce apoptosis of cardiomyocytes, oligodendrocytes, and hippocampal neurons by regulating Ca^2+^ concentration, and can also affect Ca^2+^-dependent signaling pathways, pain perception, and responses to environmental stimuli and irritating compounds** (**Earley 2012; Hu et al. 2021**)**. This channel was initially found to be expressed in sensory neurons of the dorsal root ganglion (DRG), trigeminal ganglia, and tubercle ganglia, and later researchers gradually discovered that it is expressed in nonneuronal cells (Fig. 2) such as alveoli. It was expressed in Schwann cells, epithelial cells, cardiac fibroblasts (CF), pancreatic beta cells, enterochromaffin cells, T-cells, and 105 calcitonin gene-related peptide (CGRP)- and IB4-positive neurons. In these cells, it can affect a wide range of physiological processes, mainly through the effect of Ca^2+^ influx, and the regulation of oxidative stress and neuronal peptides release (SP, bradykinin, CGRP, etc.)** (**De Logu et al. 2017; Jha et al. 2015; Meents et al. 2019; Nilius et al. 2012; Wang et al. 2019b**)**.

## *TRPA1*-Related Ligands

### TRPA1 Agonists

A variety of compounds activate *TRPA1* channels, which can be broadly divided into two categories: electrophilic activators and nonelectrophilic activators. Furthermore, *TRPA1* agonists can be classified as exogenous or endogenous (Strassmaier and Bakthavatchalam 2011). Electrophilic endogenous *TRPA1* agonists include acrolein, reactive oxygen species, while exogenous agonists of *TRPA1* include electrophiles such as allyl isothiocyanate (AITC), cinnamaldehyde (CA), and allicin. Although structurally dissimilar, they exert their activity by covalently modifying the intracellular N-terminal cysteine residue of *TRPA1*. *TRPA1* can also be activated by noncovalently modified compounds (Skerratt 2017). Reactive and nonreactive compounds may activate *TRPA1* by binding to different sites on the channel (Strassmaier and Bakthavatchalam 2011) and have different effects on a variety of diseases; for example, CA regulates blood sugar through *TRPA1*-ghrelin and other pathways (Zhu et al. 2017). In recent years, it has been suggested that CA may have a protective effect on the gastrointestinal tract by inducing the secretion of prostaglandin E2 (PGE2) (Manneck et al. 2021). In addition to the above, *TRPA1* is activated by cold, heat, and mechanical stimuli (Talavera et al. 2020). Caffeine can activate mouse *TRPA1* channels, but suppresses human *TRPA* (Nagatomo and Kubo 2008). Many traditional herbal medicines might exert their pharmacological activity through modulating the activity of *TRP* channels (Sanechika et al. 2021), including petasites or parthenolide, safranal might exert analgesic properties by partial agonism and selective desensitization of the *TRPA1* channel (Li Puma et al. 2019). The other herbal extracts, flavonoid aglycones, and glycycoumarin activated *TRPA1* (Sanechika et al. 2021).

### TRPA1 Antagonists

*TRPA1* antagonists are mainly divided into selective and nonselective antagonists; acyl-glucuronide metabolite of ibuprofen (De Logu et al. 2019), derivatives of dipyrone and pyrazolone (Nassini et al. 2015), A967079 (Chen et al. 2011), xanthine derivatives (such as HC-030031) (McNamara et al. 2007), and GDC-0334 are considered to be *TRPA1* selective antagonists (Balestrini et al. 2021); gadolinium, amiloride, gentamycin, and ruthenium red are nonselective *TRPA1* antagonists (Baraldi et al. 2010). Various antagonists have also been extensively studied in terms of diseases, Ibuprofen-acyl-glucuronide, for example, has been shown to reduce the early pain response to formalin by both local and systemic administration, this new effect may contribute to the analgesic and anti-inflammatory activity of maternal drugs (De Logu et al. 2019). Such as the treatment of allergic rhinitis with HC-030031 (Fang et al. 2021), which has also been observed to help modulate depression- and anxiety-related behaviors in mice (de Moura et al. 2014). It was also found in animal experiments that GDC-0334 inhibited allergen-induced pulmonary neurogenic inflammation through the regulation of SP (Balestrini et al. 2021).

Both agonists or antagonist of *TRPA1* might exert protective or harmful effects through different pathways. How to maximize its protective effect and reduce damage is an issue worthy of our in-depth study, research, and discussion.

## *TRPA1* and Disease

### Nervous System

#### Migraine

Migraine mainly manifests as headache, nausea, vomiting, and hypersensitivity to stimuli such as light and sound. In some cases, there are also precursors (Benemei and Dussor 2019). Migraine *aura* is currently thought to be associated with cortical spreading depression (CSD), while migraine is associated with activation of the trigeminal neurovascular system (Goadsby and Holland 2019; May and Schulte 2016). After CSD, oxidative stress spreads downstream within the trigeminal nociceptive system and may be involved in the coupling of CSD to trigeminal vasculature activation in migraine pathology (Shatillo et al. 2013). A pathophysiological link between migraine and meningeal trigeminal innervation was proposed as early as 1979 (Kleeberg-Hartmann et al. 2021). Trigeminal nociceptors that innervate the cranial dura are sensitive to chemical and mechanical stimuli, suggesting that activation of these fibers may trigger headaches (Edelmayer et al. 2012).

*TRPA1* receptor channels are localized to subpopulations of unmyelinated or thinly myelinated C- or Aδ fiber neurons in the dorsal root ganglia, trigeminal ganglia, and vagal ganglia and are almost exclusively expressed by C fibers present in the same nociceptive neuron (Benemei et al. 2014). Numerous studies have shown that meningeal *TRPA1* may mediate migraine responses to environmental stimuli, one of the most common triggers of migraine (Edelmayer et al. 2012). For example, hydrogen sulfide (H_2_S) and nitric oxide (NO) can cause headaches (Benemei and Dussor 2019) and are activated in three ways: (1) cysteine residues of *TRPA1* channels are targets of NO, and NO nitrosylation may contribute to channel sensitization (Demartini et al. 2017); (2) when H_2_S and NO combine to form nitroxyl, the two compounds work together to react with and activate *TRPA1* through covalent modification (Benemei and Dussor 2019); and (3) polysulfides (H_2_Sn) generated from the interaction between H_2_S and NO also activate *TRPA1* (Talavera et al. 2020).

How does the activation of *TRPA1* mediate pain perception? Nakamura et al. proposed that *TRPA1* activation by phosphorylating p38 mitogen-activated protein kinase releases SP from primary sensory neurons by increasing intracellular Ca^2+^ (an inflammatory response) while also inducing CGRP release from trigeminal neurons and dura mater tissue. In addition, *TRPA1* may activate oxidative stress by inducing the occurrence of intracellular Ca^2+^ overload, resulting in increased release of intracellular inflammatory factors, neuroinflammation, and migraine (Demartini et al. 2017). CGRP can act in both the periphery to enhance nociceptor sensitization and the CNS to enhance sensory input, thereby heightening pain perception (Russo 2015).

**Fig. 3:**
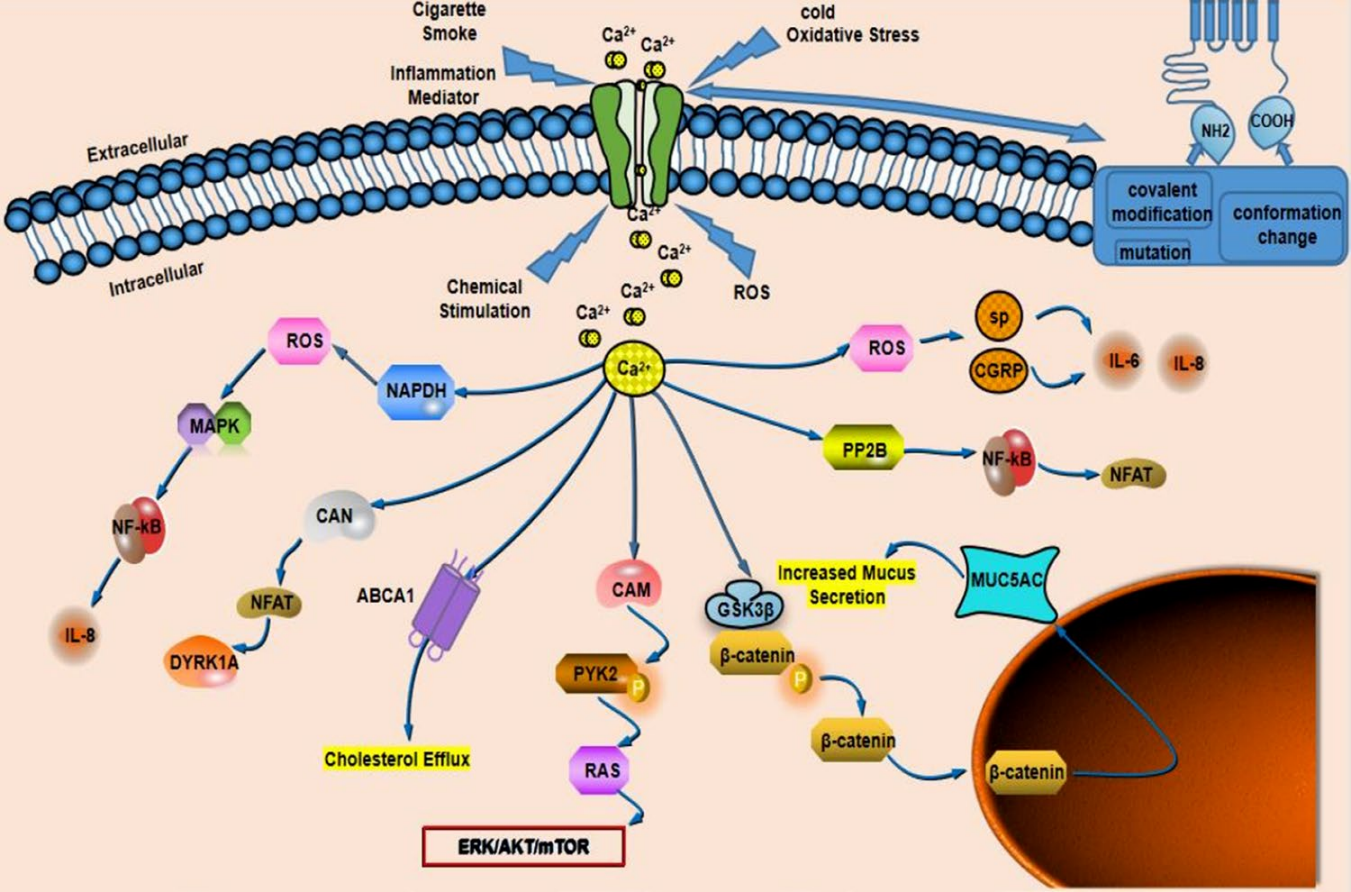
TRPA1 affects various systems and various diseases by regulating Ca^2+^ concentrations and affecting downstream-related mechanisms. It affects migraine by promoting neuropeptide release through oxidative stress; affects AD by activating PP2B signaling; and affects COPD through specific mechanisms of EGFR, GSK3β, p38 MAPK, and β-catenin. Activation of the RAS-ERK/AKT/mTOR signaling pathway affects lung cancer. TRPA1 promotes upregulation of ABCA1 and cholesterol efflux involved in the development of atherosclerosis and protects necrotic myocardium through the CaN-NFAT-DYRK1A signaling pathway. Activation of Ca^2+^-dependent enzymes and downstream transcription factors promotes T-cell activation and reduces colonic inflammation. COPD: chronic obstructive pulmonary disease. AD, Alzheimer’s disease. ROS, Reactive oxygen species. SP, substance P. CGRP, calcitonin gene‐related peptide. IL-6, interleukin-6. IL-8, interleukin-8. PP2B, serine/threonine-protein phosphatase 2B. NF-κB, nuclear factor κB. NFAT, nuclear factor of activated T-cells. mTOR, mammalian target of rapamycin. GSK-3β, glycogen synthase kinase-3β. ERK, extracellular-signal-regulated kinase. AKT, protein kinase B. MAPK, Mitogen-activated protein kinase. MU5AC, mucins 5AC. CaM, calmodulin. Pyk2, Proline-rich tyrosine kinase 2. Ras, rat sarcoma. ABCA1, ATP-binding cassette transporter A1. CaN, calcineurin. DYRK1A, dual-specificity tyrosine-regulated kinase-1a. NADPH, Nicotinamide adenine dinucleotide phosphate

*TRPA1* is sensitive to oxidative stress and is also the target of emerging drugs involved in migraine prevention. The data show that the critical role of *TRPA1* in regulating cortical susceptibility to CSD is functionally related to ROS and CGRP and that this role is the central mechanism. Because ROS trigger *TRPA1* activation and CGRP production, they create a positive feedback loop in regulating cortical susceptibility to CSD. Through this pathway, ROS promote CSD dissemination for the subsequent development of migraine. Therefore, ROS/*TRPA1*/CGRP signaling contributes to CSD induction (Jiang et al. 2019) (Fig. 3). Efficacy of monoclonal antibodies against CGRP or its receptor (calcitonin receptor-like receptor/receptor activity modifying protein-1, CLR/RAMP1) implicates peripherally released CGRP in migraine pain. CLR/RAMP1 activation in human and mouse Schwann cells generates long-lasting signals from endosomes that evoke cAMP-dependent formation of NO. NO, by gating Schwann cell *TRPA1*, releases ROS, which in a feed-forward manner sustain allodynia via nociceptor *TRPA1* (De Logu et al. 2022).

About “Headaches” another neurological disease that affects us in the Lenovo—Progressive Multiple Sclerosis syndrome, but the mechanism is unclear. It is only known that an endogenous agonist of *TRPA1* may sensitize *TRPA1* in trigeminal nerve Nociceptor to trigger periorbital mechanical allodynia (Dalenogare et al. 2021).

#### Alzheimer’s Disease (AD)

AD is a type of neurodegenerative disease characterized by age-related cognitive and functional decline (Scheltens et al. 2021; Soria Lopez et al. 2019). Historically, AD was characterized by β-amyloid or Aβ from a pathological point of view (Dugger and Dickson 2017), but the current focus has expanded to include responses in other cell populations, such as microglia-mediated inflammation, which has taken center stage in functional studies of the pathogenesis of the disease (Scheltens et al. 2021).

In 2012, it was first reported that *TRPA1* was expressed in astrocytes in the superficial layer of rat trigeminal caudal nucleus (Lee et al. 2012). Astrocytes are the most abundant cells in the central nervous system (CNS). These cells are able to transport ions, absorb neurotransmitters, and produce neurotrophic factors to maintain CNS function and homeostasis; among them, astrocyte ion channel plays a key role (Ikeshima-Kataoka 2016; Verkhratsky and Nedergaard 2018). Studies have shown that *TRPA1* in astrocytes can be activated by ROS, NO, inflammatory factors, and pathological markers of neurodegenerative diseases such as Aβ, and it induces the inward flow of Ca^2+^ in astrocytes (Wang et al. 2022). Increased Ca^2+^ influx is a key event in the activation of serine/threonine-protein phosphatase 2B (PP2B) signaling and astrocyte inflammation (Lee et al. 2016). Imbalance of Ca^2+^ concentration leads to excessive activation of astrocytes, which in turn releases proinflammatory factors leading to neurodegeneration. Activation of nuclear factor (NF)-κB, PP2B, and nuclear factor NFAT, which activates T-cells, leads to an inflammatory response (Wang et al. 2022), thereby promoting the development of AD.

In the APP/PS1-21 mouse model of AD, blocking *TRPA1* normalized astrocyte activity, avoided peri-synaptic astrocytic process regression, prevented neuronal dysfunction, and maintained structural synaptic integrity (Paumier et al. 2022). This indicates that the loss of *TRPA1* channel function hinders the progression of AD. In summary, the activation and inhibition of *TRPA1* channels have different effects on astrocytes. *TRPA1*-Ca^2+^-PP2B signaling may play a key role in regulating astrocyte-derived inflammation and AD pathogenesis (Lee et al. 2016). TRPA1 channels emerge as potential therapeutic targets for promoting neuroprotection.

#### Peripheral Neuropathy

Peripheral neuropathy here mainly includes chemotherapy-induced peripheral neuropathy (CIPN) and diabetic peripheral neuropathy (DPN). CIPN and its associated pain are a major side effect of some chemotherapy drugs used in cancer treatment (Moore et al. 2018). The chemotherapeutic drug oxaliplatin or one of its metabolites, oxalate, can inhibit the prolyl hydroxylase-mediated hydroxylation of an N-terminal proline residue of *TRPA1*, which induces *TRPA1* sensitization to ROS and endows *TRPA1* with cold sensitivity via transduction of ROS signaling (Nakagawa and Kaneko 2017). In a streptozotocin-induced diabetic mouse model, early cold hypersensitivity in DPN is mediated through *TRPA1* sensitization during diabetic vascular injury (Hiyama et al. 2018). ROS play an important role in maintaining pain in neuropathic pain models. Selective ROS scavenging at the peripheral or central level, respectively, inhibits the corresponding *TRPA1* components, thus inhibiting oxidative stress against *TRPA1* contributes to the attenuation of mechanical allodynia (De Logu et al. 2020b). Schwann cell *TRPA1* is required to coordinate neuroinflammation and oxidative stress to maintain neuropathic pain in complex areas of pain caused by ischemia–reperfusion (De Logu et al. 2020a). It has also been suggested that inhibition of *TRPA1* activity by activation of AMPK, a ubiquitously expressed serine/threonine kinase, is a useful factor in the prevention of diabetic neuropathy (Wang et al. 2018). Overall, therapeutic strategies targeting *TRPA1* for the treatment of painful peripheral neuropathy as well as various peripheral ischemic diseases such as peripheral arterial occlusive disease may be warranted (Hiyama et al. 2018).

### Respiratory System

#### Asthma

Asthma is a respiratory disease characterized by airway inflammation, airflow obstruction, and airway hyperresponsiveness, mainly manifesting as cough, dyspnea, and wheezing (Balestrini et al. 2021). Although inflammation and the immune system play key roles in the pathogenesis of asthma, the efficacy of anti-inflammatory agents is limited, especially in patients with hormone-refractory asthma, suggesting that noncanonical pathways and cellular components are involved in disease manifestations.

*TRPA1* is an ideal sensor for airway stimuli, is mainly expressed in small diameter nociceptive neurons, and is required for cigarette smoke (CS)-induced airway inflammation (Andrè et al. 2008). There are complex interactions between airway cells and nerve fibers. In humans, *TRPA1* polymorphisms are associated with reduced asthma control (Balestrini et al. 2021). Based on the comprehensive review of relevant literature, the author believes that the effect of *TRPA1* on asthma is mainly reflected in three aspects: participating in the inflammatory response, mediating the change of cough, and participating in the process of asthma exacerbation.

Caceres et al. recently showed that in a mouse model of allergic asthma, inhaled stimuli may activate *TRPA1* expressed in sensory neurons innervating the airways, mediate inflammatory leukocyte infiltration and enhance pulmonary mucus production and airway hyperresponsiveness (Engel et al. 2011). The released neuropeptides induce bronchoconstriction, vasodilation, immune cell recruitment, and modulation of the inflammatory response. These effects promote protective physiological responses such as coughing, increased mucus secretion, and shallow breathing (Wu et al. 2021a). *TRPA1* is an ion channel that mediates cough signaling (Luostarinen et al. 2021), which is key to the diagnosis and treatment of asthma. Cough is one of the important manifestations of asthma. The main types of fibers that cause cough are C fibers and Aδ fibers. The former has a slower conduction velocity, is unmyelinated, and is chemically sensitive, while the latter is myelinated, has fast conduction, and is more sensitive to mechanical stimuli (Bonvini and Belvisi 2017). Moreover, the airways have elevated levels of PGE2 and bradykinin (Choudry et al. 1989), both of which have been shown to activate *TRPA1* (Grace et al. 2012) and cause cough (Choudry et al. 1989; Grace et al. 2014; Maher et al. 2009).

Th1/Th2 imbalance is the cause of allergic asthma (Li et al. 2019a). Allergic inflammation is driven by the activity of Th2 cytokines, which enhance *TRP* channels through multiple mechanisms under different inflammatory conditions (Meng et al. 2021). With the increase in asthma morbidity and mortality, asthma exacerbated by environmental pollution has attracted increasing attention. Trimellitic anhydride (TMA), a typical pollutant that is ubiquitous in the atmosphere, is a typical low-molecular-weight chemical sensitizer that can induce typical Th2 responses. OVA plus TMA-treated mice showed higher *TRPA1* gene and protein expression and Th2 cytokine levels. Interestingly, similar results were detected in mice treated with OVA plus PM2.5 (Li et al. 2020a). This shows that environmental pollutants such as TMA can aggravate asthma (Li et al. 2019a), and this process may be related to the activation of the *TRPA1* channel.

#### Chronic Obstructive Pulmonary Disease (COPD)

COPD is a persistent respiratory symptom caused by abnormalities of the airways and/or alveoli, such as coughing, wheezing, and airflow limitation, usually due to high exposure to toxic particles or gases (Soriano et al. 2018). *TRPA1* has been identified as a pro-tussive receptor in both clinical trials and guinea pig models, and this effect can be blocked by selective antagonists (Belvisi et al. 2011). Therefore, *TRPA1* not only has an impact on the pathogenesis of asthma but also may be related to the neurogenic mechanism of bronchitis and COPD in different stages. Both asthma and COPD cause airway obstruction and are associated with chronic inflammation of the airways. However, the nature and location of inflammation differ between the two, resulting in different pathologies, clinical manifestations, and responses to therapy (Barnes 2017). In summary, the effects of *TRPA1* on the pathogenesis of COPD were mainly analyzed from the aspects of regulating airway mucus, bronchoconstriction, and inflammation.

Mucus hypersecretion is a pathological feature of acute inflammation and COPD (Memon et al. 2020). *TRPA1* mediates CS-induced bronchial and alveolar epithelial cell injury by regulating airway mucus secretion, inflammation, smooth muscle contraction, and mitochondrial damage (Wang et al. 2019a). Studies have shown that exposure of primary human bronchial epithelial cells and mice to pine wood smoke particulate matter results in increased cytosolic calcium, increased phosphorylation of GSK3β, and increased nuclear entry after dephosphorylation of β-catenin owing to activation of *TRPA1*, thereby regulating the increased expression of mucin 5AC (MUC5AC), which eventually leads to an increase in mucus secretion (Memon et al. 2020). *TRPA1* activation and intracellular Ca^2+^ can disrupt cellular integrity and cause EGFR ligand shedding and β-catenin accumulation in the cytoplasm, perinuclear region, and nucleus, in addition to being associated with the regulation of *TRPA1* and EGFR downstream signaling. These effects occur through specific mechanisms involving *TRPA1*, EGFR, GSK3β, p38 MAPK, and β-catenin (Memon et al. 2020). Furthermore, *TRPA1* channel-mediated Ca^2+^ release in endolysosomes directly triggers vesicular exocytosis and CGRP release, greatly enhancing DRG neuronal excitability. Thus, in addition to acting through Ca^2+^ influx, *TRPA1* channels also trigger vesicle release in sensory neurons by releasing Ca^2+^ from lysosome-like organelles (Shang et al. 2016). Taken together, both epithelial and neuronal *TRPA1* may be important in both nonallergic and allergic mechanisms leading to abnormal mucus hypersecretion in humans.

In recent years, nonneuronal localization of functional *TRPA1* receptors has been demonstrated in muscle cells of airway smooth muscle (ASM) bundles** (**Wang et al. 2019b**).** ASM contraction controls airway caliber and can coordinate airway inflammation and remodeling (Jha et al. 2015). CS or extract exposure rapidly activates Ca^2+^ influx through *TRPA1* into human airway smooth muscle cells, resulting in phosphorylation of myosin light chains and regulation of ASM contractility (Spix et al. 2022). The *TRPA1* channel may be a determinant of ASM contraction, part of a novel mechanism that controls (pathological) airway, and ASM physiology (Jha et al. 2015).

It is well known that *TRPA1* may be a key gatekeeper in regulating inflammatory responses to stimuli, including bacterial endotoxins, environmental irritants or inflammatory mediators (Lee et al. 2016). The researchers used the *TRPA1*-specific antagonist HC-030031 to abolish neuropeptide-mediated constriction of isolated guinea pig bronchial segments after perfusion with α, β-unsaturated aldehydes or CSE; in contrast, in *TRPA1*-deficient mice, instillation of CSE into the trachea failed to trigger neurogenic plasma extravasation. This suggests that the interaction of electrophilic components in CS with *TRPA1* is the main mechanism by which CS induces acute neurogenic inflammation of the airways (Bessac and Jordt 2008).

#### Lung Cancer

There are also many established links between cancer and ion channels, and *TRP* channels are also oncogenic intracellular ion channels (Grimm et al. 2018). Among all *TRP* channels, *TRPA1* was the most highly upregulated in lung squamous cell carcinoma and was the second most highly upregulated in lung adenocarcinoma (LUAD) (Takahashi et al. 2018). It has been shown that using agonists and antagonists of *TRPA1* channel, respectively, can significantly increase and decrease Ca^2+^ levels in cancer cells, thus affecting the initiation and development of apoptosis (Özkal and Övey İ, 2020).

*TRPA1* increases the phosphorylation of proline-rich tyrosine kinase 2 (PYK2), can be elevated by [Ca^2+^] via calmodulin (CaM) and can activate the RAS-ERK/AKT/mTOR signaling pathway (Cullen and Lockyer 2002), which mediates Ca^2+^ influx through Ca^2+^-CaM-mediated oxidative conditions. PYK2 activation amplifies the prosurvival/antiapoptotic signaling pathway (Takahashi et al. 2018). *TRPA1* has also been shown to accelerate metabolism in cancer cells that require high concentrations of reactive oxygen species to maintain their high proliferation rates (Sosa et al. 2013). Oxidative stress defense is particularly important for cancer cells to gain anchorage independence, and *TRPA1* channels can be directly activated by oxidants/electrophiles through cysteine modification and have the highest oxidative sensitivity (De Logu et al. 2021b; Takahashi et al. 2018). *TRPA1* induces Ca^2+^ influx in response to ROS generated in the inner cells of tumor spheroids and protects them from apoptotic death (Takahashi et al. 2018).

With regard to LUAD, it has been demonstrated that the membrane receptor fibroblast growth factor receptor 2 (FGFR2) is a key driver of disease progression. FGFR2 recruits proteins to a proline-rich motif at the C-terminus of *TRPA1*, resulting in receptor phosphorylation and subsequent activation of downstream signaling pathways that promote LUAD progression and showing that astrocytes antagonize brain metastases by mediating the downregulation of *TRPA1* via exosome-delivered miRNA-142-3p (Berrout et al. 2017). Furthermore, it has been found that elimination of Schwann cell *TRPA1* attenuates lung cancer-induced mechanical allodynia, and therefore, Schwann cell *TRPA1* may represent a potential target for the treatment of cancer pain (De Logu et al. 2021a).

### Circulatory System

#### Atherosclerosis

Atherosclerosis, characterized by arterial wall hardening and arterial lumen narrowing, is thought to be a ROS-induced low-density lipoprotein (LDL) chronic inflammatory disease caused by oxidation (Wang et al. 2019b; Zhao et al. 2016). Ion channels play a key role in vascular disease (Wang et al. 2020); among them, the activation of the *TRPA1* channel has a protective effect on the development of atherosclerosis (Wang et al. 2019b), and its dependent antiatherosclerotic effect may require the cooperation of multiple physiological pathways: regulating the phenotypic plasticity of macrophages, mediating ROS, and participating in vascular inflammation in the pathophysiological process of atherosclerosis (Moriya 2019), while macrophages are an important component in inflammatory infiltration and are present in all stages of the disease (Bartlett et al. 2019).

Macrophages are classified into the M1 and M2 types; M2 macrophages are atheroprotective and show anti-inflammatory effects, while M1 macrophages are largely involved in the expansion and progression of atherosclerotic lesions (Bartlett et al. 2019). Qiang Wang et al. demonstrated by RNA-seq that *TRPA1* can regulate H3K27me3 (closed chromatin whose modification is regulated by PRC2) by protecting EZH2 (histone methyltransferase-active protein, one of the subunits of PRC2) from degradation regulation) to inhibit macrophage activation. *TRPA1* deficiency leads to EZH2 degradation and chromatin opening, promoting M1 macrophage-associated gene transcription and atherosclerotic plaque formation (Wang et al. 2020). In response to atherosclerotic stimulation, *TRPA1* is activated, and vascular cells release ROS, which stimulate smooth muscle cell migration and collagen deposition, leading to the development of atherosclerotic plaques (Kattoor et al. 2017).

The initial event in the development of atherosclerosis is endothelial injury. This causes infiltration into and accumulation of LDL cholesterol in the subendothelial space (Kattoor et al. 2017). LDL becomes oxidized to form oxidized LDL (ox-LDL) in pathologic states (Ketelhuth and Hansson 2011).The expression of *TRPA1* in atherosclerotic lesions mainly occurs in the foam cell area of macrophages, and the most important atherosclerotic molecule, ox-LDL, increases the intracellular Ca^2+^ level through *TRPA1* in macrophages and promotes the upregulation of ATP-binding cassette transporter A1 (ABCA1) and cholesterol efflux, suggesting that macrophage *TRPA1* may be involved in atherogenic molecule-induced dysregulation of cholesterol metabolism and inflammation and the development of atherosclerosis through its influx of Ca^2+^ (Zhao et al. 2016).

#### Myocardial Infarction (MI)

MI is defined as sudden ischemic death of myocardial tissue. Clinically, MI is usually a thrombotic occlusion of a coronary vessel due to rupture of a vulnerable plaque. Ischemia causes severe metabolic and ionic perturbations in the affected myocardium and results in a rapid depression of contractile function (Frangogiannis 2015). *TRPA1* is located in the sarcolemma and intercalated discs in cardiomyocytes (Üstünel and Özgüler 2021) and contributes to acrolein-induced calcium overload and hypercontraction (Conklin et al. 2019). Inhibition of *TRPA1* promotes angiogenesis after MI, thereby attenuating myocardial ischemic injury through a mechanism of inhibition of phosphatase and tensin homolog expression and subsequent activation of PI3K/Akt signaling (Li et al. 2020b), suggesting that ischemia‒reperfusion (I/R) activation of *TRPA1* exacerbates MI; this channel may be a potential target for alleviating I/R injury (Conklin et al. 2019).

After MI, physiological compensatory mechanisms promote cardiomyocyte loss and fibrosis through pathological remodeling. CF is a key cellular component of left ventricular remodeling after MI and is a major contributor to cardiac fibrosis. After MI stimulation, CFs are activated to proliferate, differentiate into cardiac myofibroblasts (CMFs), and play an important role in the fibrotic healing response (Hao et al. 2019; Li et al. 2019b). Methylglyoxal (MG, a highly active dicarbonyl compound) activates *TRPA1* with deleterious cardiovascular effects associated with the activation of fibrosis. Inhibition of *TRPA1* can reduce MG-induced Ca^2+^ influx, inhibit MG-induced fibroblast proliferation, and increase α-smooth muscle actin expression (Wang et al. 2019b). In an in vitro study, surprisingly, *TRPA1* overexpression fully activated CMF transformation, while CF lacking *TRPA1* induced transdifferentiation to transforming growth factor β- (TGF-β-), which promotes the Ca^2+^-responsive activation of calcineurin (CaN). Furthermore, dual-specificity tyrosine-regulated kinase 1a (DYRK1A) regulates CaN-mediated nuclear translocation of NFAT and *TRPA1*-dependent transdifferentiation. In summary, *TRPA1* promotes the differentiation of CMFs after myocardial infarction injury through the CaN-NFAT-DYRK1A signaling pathway, thereby exerting a protective effect on the heart (Li et al. 2019b).

### Digestive System

#### Appetite, Taste

Taste, which includes sweet, bitter, umami, salty, and sour, induces changes in Ca^2+^ levels, pH, and/or membrane potential in taste cells of the tongue and/or neurons that transmit and decode taste signals to the brain (von Molitor et al. 2020). These taste qualities are detected by distinct subsets of cells in the taste buds. Three distinct taste receptor cell subsets within taste buds are characterized as type I to type III cells. Type III cells express the *TRP* cation channel and the acid-sensing H^+^ channel otopetrin-1, among others (Rhyu et al. 2021). In many cases, *TRP* channels function directly as receptors for certain chemosensory stimuli (Aroke et al. 2020). *TRPA1* is the Drosophila melanogaster ortholog of the human stimulatory sensor (Kang et al. 2010) and represents the first *TRP* required in separate chemosensory and thermosensory receptor cells, which function in taste and temperature discrimination (Kim et al. 2010). Drosophila responses to higher levels of bitter compounds are mediated by direct activation of *TRPA1* (Leung et al. 2020), and deletion of *TRPA1* results in reduced oral sensory sensitivity to menthol in mice (Lemon et al. 2019).

*TRPA1* is also a structurally related thermosensitive cation channel, and the coexpression of this calcium-conducting *TRP* channel with CGRP in oral trigeminal C and Aδ fibers has been clearly reported (Kichko et al. 2018). *TRPA1* is expressed in taste receptor neurons that respond to aversive compounds (Kim et al. 2010) and can be indirectly affected by the release of substance P and CGRP from trigeminal neurons; their subsequent effects on CGRP receptors expressed in type III taste receptor cells affect some but not all primary taste qualities (Rhyu et al. 2021).

Kazuaki Ohara et al. found that *TRPA1* channels are involved in β-eudesmol (an oxygenated sesquiterpene present in medicinal or edible plants) regulation of feeding behavior, since there is a *TRPA1*-derived gastric vagal nerve activity (Ohara et al. 2017). In addition, single nucleotide polymorphisms in *TRPA1* have been shown to affect and modulate chemosensation and taste.

#### Pancreatic Cancer

Pancreatic cancer is one of the leading causes of cancer-related deaths worldwide. Despite advances in early detection and treatment, the prognosis remains dismal (Manrai et al. 2021). Aberrant expression and/or activity of ion channels may lead to malignant transformation and tumor progression. *TRP* channels have been shown to play a role in Pancreatic cancer biology (Shi et al. 2022), *TRPA1* is expressed in the pancreatic ductal adenocarcinoma cell (PDAC) cell lines Panc-1, MIA Paca-2, and BxPC-3 (Cojocaru et al. 2021), and it is located at the interface between the intracellular and extracellular spaces in the cell membrane, sensing and modifying the tumor microenvironment; this in itself is a driver of the aggressiveness of PDAC (Hofschröer et al. 2020).

Relevant information on the impact of *TRPA1* on Pancreatic cancer is relatively limited. According to the existing reports, the summary is as follows.

First, researchers have identified that silencing *TRPA1* expression induces a significant increase in migration potential and that *TRPA1* channels use pore-independent signaling pathways to participate in migration (Cojocaru et al. 2021).

Second, nonselective cation currents were activated by AITC in Panc-1 cells and inhibited by the selective *TRPA1* antagonist A-967079 (Cojocaru et al. 2021), indicating that the cation current is regulated by *TRPA1*. It is well known that calcium signaling drives key oncogenic processes such as proliferation, migration, invasion, and angiogenesis (Kutschat et al. 2021). During proliferation, Ca^2+^ has a fundamental role in cell cycle initiation and progression, as evidenced by the inhibition of several Ca^2+^ channels leading to cell cycle arrest (Mesquita et al. 2021). Based on the above information, we infer that *TRPA1* may affect the cell cycle of PDAC through the regulation of Ca^2+^, but this possibility remains to be further studied and explored.

Finally, cancer cell survival depends on oxidative stress defense against ROS that accumulate during tumorigenesis. Although there is currently no evidence that *TRPA1* is directly involved in the process of Pancreatic cancer oxidative stress, there are data showing that NRF2, an oxidant defense transcription factor, directly controls the expression of *TRPA1*; more notably, the KEAP1-NRF2 pathway, which plays a central role in protecting cells from oxidative stress by inducing ROS-neutralizing gene expression, was shown to stimulate carcinogenesis and support tumor maintenance in Pancreatic cancer (Takahashi et al. 2018). Taken together, these findings indicate that *TRPA1* channels in PDAC cell membranes regulate cellular processes through pore-dependent and pore-independent mechanisms (Cojocaru et al. 2021). The putative role of endogenous channels and activators opens new perspectives to study TRPA1 in PDAC and nontumor cell lines, and TRPA1 channels are promising as PDAC antitumor therapy (Cojocaru et al. 2021).

#### Inflammatory Bowel Disease

Inflammatory Bowel Disease refers to ulcerative colitis (UC) and Crohn’s disease (CD) and is characterized by chronic idiopathic inflammation (Sairenji et al. 2017). In addition to genetic susceptibility, there is also a certain relationship with the immune response (Zhang and Li 2014). *TRPA1* has a broad tissue distribution, and *TRPA1* mRNA and protein were also detected in intestinal epithelial cells (Bertin et al. 2017). *TRPA1* is also reported to be upregulated in Inflammatory Bowel Disease patients and plays a crucial role in the inflammatory response of Inflammatory Bowel Disease (Wu et al. 2021a).

As a mechanosensor, activation of *TRPA1* is thought to be a factor in the generation of afferent mechanical hypersensitivity in models of colitis. *TRPA1* is also activated by the release of inflammatory mediators when tissue is damaged or diseased (Hassan et al. 2020). TRPs essentially control the vesicular exocytosis of CGRP by virtue of their inherent Ca^2+^ conductance, nicotinic acetylcholine receptor channels depolarize nerves, and the support of voltage-gated calcium channels is then required to trigger CGRP release (Kichko et al. 2018). Meanwhile, upregulation of *TRPA1* at the level of primary sensory neurons also contributes to the release of SP and CGRP. Inhibition of *TRPA1* thus reduces the inflammatory process by reducing colonic neuropeptide (substance P and CGRP) release from sensory neurons outside the gut. In addition, *TRPA1* was also detected in CD4^+^ T-cells infiltrating colon tissue samples from patients with UC and CD (Landini et al. 2022), and *TRPA1* is involved in the control of CD4^+^ T-cell activation and proinflammatory responses in two different T-cell-mediated colitis models (Bertin et al. 2017). Among immune cells in the gut, T-cells play an important role in maintaining gut immunity and homeostasis (Wu et al. 2021c), and immune cells are heavily dependent on the Ca^2+^ signaling pathway. For example, antigen recognition by T-cell receptors results in IP3-dependent release of Ca^2+^ from the endoplasmic reticulum. This reduction in endoplasmic reticulum Ca^2+^ levels results in the activation of Ca^2+^ release-activated Ca^2+^ channels in the membrane and the influx of extracellular Ca^2+^. This in turn activates Ca^2+^-dependent enzymes and downstream transcription factors, such as NF-κB and NFAT, which then lead to T-cell activation (Naert et al. 2021). Thus, *TRPA1* in CD4^+^ T-cells appears to reduce the severity of T-cell-mediated colitis.

Intestinal fibrosis is a common complication of Inflammatory Bowel Disease, affecting 30%–50% of CD patients. It is characterized by the accumulation of myofibroblasts and excessive deposition of extracellular matrix (Hirota 2018). However, in some clinical studies, elevated levels of TGF-β1 mRNA were found in the intestinal mucosa of CD and UC patients, especially in the lamina propria region of immune cells and myofibroblasts. Combining this fact with our own findings in human surgical samples (i.e., coaccumulation of *TRPA1*-/HSP47- double-positive myofibroblasts in stenotic areas) (Hiraishi et al. 2018), we speculate that *TRPA1* not only has a protective effect on the intestine but also has a certain effect on intestinal fibrosis caused by intestinal by targeting the *TRPA1* signaling axis of myofibroblasts.

### Urinary System

#### Acute Kidney Injury

The causes of Acute kidney injury are traditionally divided into three categories: prerenal, renal (with direct intrinsic renal injury), and postrenal. Approximately, two-thirds of acute tubular necrosis is caused by renal ischemia‒reperfusion injury (IRI) or sepsis, and one-third is caused by direct or indirect nephrotoxicity (Lameire et al. 2013). However, the author believes that oxidative stress and inflammation play an important role in the pathophysiology of renal ischemia‒reperfusion or other causes of nephrotoxicity.

*TRPA1* expression was significantly increased in renal tubular epithelial cells both in patients with Acute kidney injury and in an in vitro model (under hypoxia-reoxygenation (H/R) conditions). During the reperfusion phase of IR, excess ROS are generated, leading to increased oxidative stress in renal tissue. In addition to being sensitive to ROS, *TRPA1* is also highly permeable to Ca^2+^. H/R induced ROS-dependent *TRPA1* activation, which increased intracellular Ca^2+^ levels, increased NADPH oxidase activity, activated MAPK/NF-κB signaling, and promoted the release of the inflammatory factor IL-8. Therefore, tubular *TRPA1* is a sensor of oxidative stress and a key regulator of activated signaling pathways (Wu et al. 2021b). Similar to IRI, ROS also activate MAPK and NF-κB signaling in cisplatin (DDP, a commonly used chemotherapeutic drug)-induced nephrotoxicity. More importantly, treatment of HEK293 cells with the *TRPA1* antagonist HC-030031 reduced the expression of phosphorylated IκBα, IKKβ, JNK, ERK, and p38. *TRPA1* regulates the phosphorylation of the MAPK/NF-κB signaling pathway, promotes the production and release of inflammatory cytokines and mediators, and mediates DDP-induced cellular inflammation and apoptosis through the MAPK/NF-κB signaling pathway (Yuan et al. 2021).

In addition, *TRPA1* in renal tubular epithelial cells was identified to be expressed in macrophages, and the role of macrophages in IRI cannot be underestimated. The *TRPA1* activator AITC was able to attenuate macrophage activation and foam cell formation (Ma et al. 2019); the same pathway mediates H/R injury in vitro and prevents Ang-II-induced renal injury and ischemia‒reperfusion renal injury in vivo by maintaining mitochondrial hemostasis and downregulating macrophage-mediated inflammatory responses (Ma et al. 2019; Wu et al. 2021a).

#### Lower Urinary Tract Dysfunction

Lower Urinary Tract dysfunction is a common sequelae of neurological disorders. While it is mostly not fatal, the associated social disturbances, especially reduced quality of life, should not be underestimated (Franken et al. 2014; Panicker et al. 2015). In the past 20 years, *TRP* channels have become increasingly important in this field of research (Franken et al. 2014), with important sensory functions in lower urinary tract symptoms (LUTs) (Deruyver et al. 2015), because *TRPA1* activation triggers pain have been demonstrated in human urothelial cells and C-fiber afferents in the lamina propria and detrusor muscle, and a role for *TRPA1* in afferent and efferent sensory signaling in human outflow regions has been suggested. Intravesical *TRPA1* activators can initiate detrusor overactivity, pain perception (suprapubic pain, dysuria…), and/or heat sensitivity (“burning” sensation, bladder cooling reflex). Alternatively, these proteins can be targeted to alter sensory nerve function (excitability), and a role in sensory transduction in LUTs has been supported by animal experiments (Andersson 2019; Deruyver et al. 2015).

The possibility that the bladder is innervated by at least two afferent nerves has been proposed: type A expresses the *TRPA1* receptor, which induces PGE release and excites the detrusor, and type B expresses the *TRPV1*, *TRPA1*, and *TRPC* receptors and releases CGRP that inhibits the detrusor (Daugherty et al. 2021). Increased expression of *TRPA1* in the bladder wall is associated with the establishment of overactive bladder and lower urinary tract symptoms, *TRPA1* activation triggers pain, protective reflexes, and local release of peripheral neurotransmitters and is associated with spontaneous and involuntary bladder contractions in spinal cord injury (Blaha et al. 2019; Wu et al. 2021a). The effect of its activation on bladder contractility has been attributed to stimulation of *TRPA1*-expressing sensory nerve fibers causing them to release SP and PGE2, each capable of activating contractile cells through tachykinin and PGE receptors on the surface of detrusor smooth muscle (Philyppov et al. 2016). Studies have also shown that HC-030031 treatment reduces the number and magnitude of nonvoiding contractions (NVCs), and inhibition of *TRPA1* can effectively reduce bladder activity; *TRPA1* antisense oligonucleotide treatment normalized spontaneous phase activity and reduced CA-induced bladder contractions and NVC numbers in spinal cord injury rats** (**Andrade et al. 2011**).**

### Others

#### Retinal Damage

I/R damage underlies many retinal diseases, such as glaucoma, diabetic retinopathy, and central retinal artery occlusion, a leading cause of visual impairment or blindness (Wan et al. 2020). Low-level ROS production, mainly by mitochondria, is necessary for the maintenance of physiological functions; however, ROS can also be dangerous. For example, oxidative stress caused by excess reactive oxygen species can lead to retinal ganglion cell death** (**McMonnies 2018**)**. When blood supply is re-established after prolonged ischemia, local inflammation and production of reactive oxygen species increase, leading to secondary injury (Wu et al. 2018). Studies have found that human retinal cells express *TRPA1* (mRNA and protein). Genetic deletion or pharmacological blockade of *TRPA1* attenuated I/R-induced increases in infiltrating macrophage numbers and levels of the oxidative stress biomarker 4-hydroxynonenal and the apoptosis biomarker active caspase-3. These findings suggest that *TRPA1* mediates oxidative stress load and inflammation that lead to retinal cell death in mice; inhibition of *TRPA1*-dependent pathways may also alleviate glaucoma-related retinal damage (Souza Monteiro de Araúja et al. 2020a).

#### Melanoma

Melanoma is a type of skin cancer caused by malignant tumors of melanocytes (Ahmed et al. 2020). *TRPA1* has been found in melanocytes and keratinocytes (Chen and Hackos 2015). On this basis, it was also found that treatment of keratinocytes with the selective *TRPA1* agonist icilin increased the expression of genes involved in cell adhesion and extracellular matrix protein synthesis (Maglie et al. 2021). During melanoma formation, macrophages, especially tumor-associated macrophages and ROS, are involved in all stages of melanogenesis (Chen et al. 2019; De Logu et al. 2021b). ROS released by infiltrating M2 macrophages may target *TRPA1*-expressing melanoma cells to amplify oxidative stress signals that affect tumor cell survival and proliferation. It can therefore be said that *TRPA1* acts as an oxidative stress sensor and amplifier, contributing to cancer progression and metastasis (De Logu et al. 2021b). Second, *TRPA1* drug blockade also reduced dacarbazine-induced nociception in a melanoma tumor-associated pain model, suggesting that this receptor may be a pharmacological agent for chemotherapy-induced pain syndrome in cancer patients receiving dacarbazine antitumor therapy (Brusco et al. 2020).

#### Diabetes

Diabetes mellitus is a series of metabolic disorder syndromes, including protein, fat, and electrolytes, caused by the absolute or relative insufficient secretion of insulin and the decreased sensitivity of target tissue cells to insulin, with hyperglycemia as the main sign. *TRP* channels play an important role in mediating glucose-stimulated insulin release by causing depolarization of pancreatic β cells and closure of KATP channels (Adhya and Sharma 2019).

*TRPA1* stimulates insulin secretion in diabetic beta cells and improves hyperglycemia. It has been reported that dual agonists of the *TRPA1*/GPR-119 receptor in intestinal STC-1 cells induce the cells to secrete glucagon-like peptide-1 (GLP-1). The released GLP-1 then causes the secretion of insulin release by acting on the GLP-1 receptor in beta cells (Bae and Sun 2011). In fact, *TRPA1* agonists themselves can also activate mouse enterocytes to release GLP-1 (Adhya and Sharma 2019). Furthermore, in mouse beta cells and INS-1 cells, catechol estrogens activate *TRPA1* channels, increase [Ca^2+^]i, and stimulate insulin secretion in a glucose-dependent manner. These effects were inhibited by pharmacological inhibitors of *TRPA1* and siRNA (Islam 2020). In addition, the antidiabetic drug glyburide has also been reported to activate the *TRPA1* channel, which may explain its antidiabetic effect as well as its ability to block KATP channels (Adhya and Sharma 2019).

## Conclusions

From our exposition of the pathogenesis of *TRPA1* involved in various diseases, it can be seen that the current research on the effect of this channel on some diseases is still in its infancy, and the research on the role of *TRPA1* channel is still complex and arduous, but we have also some gains. It was found that the effects of some different diseases also have some commonalities. The participation of the *TRPA1* channel is a key mechanism for the occurrence and development of certain diseases. These include the release of CGRP and SP in pain (headache), inflammation (colitis), and appetite regulation. For example, the regulation of oxidative stress in the pathogenesis of cancer (lung cancer, pancreatic cancer), blood vessels (atherosclerosis), and ischemia‒reperfusion (kidney injury) are mostly achieved through the regulation of Ca^2+^. In conclusion, *TRPA1* has emerged as a central therapeutic target for the treatment of multiple pathologies with a common etiology, offering an attractive therapeutic possibility for multiple diseases.
